# From molecular spectra to a density shift in dense Rydberg gases

**DOI:** 10.1038/ncomms5546

**Published:** 2014-08-01

**Authors:** A. Gaj, A. T. Krupp, J. B. Balewski, R. Löw, S. Hofferberth, T. Pfau

**Affiliations:** 1 5. Physikalisches Institut and Center for Integrated Quantum Science and Technology, Universität Stuttgart, 70569 Stuttgart, Germany

## Abstract

In Rydberg atoms, at least one electron is excited to a state with a high principal quantum number. In an ultracold environment, this low-energy electron can scatter off a ground state atom allowing for the formation of a Rydberg molecule consisting of one Rydberg atom and several ground state atoms. Here we investigate those Rydberg molecules created by photoassociation for the spherically symmetric *S*-states. A step by step increase of the principal quantum number up to *n*=111 enables us to go beyond the previously observed dimer and trimer states up to a molecule, where four ground state atoms are bound by one Rydberg atom. The increase of bound atoms and the decreasing binding potential per atom with principal quantum number results finally in an overlap of spectral lines. The associated density-dependent line broadening sets a fundamental limit, for example, for the optical thickness per blockade volume in Rydberg quantum optics experiments.

## Abstract (web summary)

Ultracold Rydberg atoms — atoms with highly excited electrons — can form molecules with ground state atoms. By tuning the principal quantum number of the Rydberg state, Gaj *et al.* study the transition from resolvable molecular lines to the mean shift regime, where indistinguishable lines form a band.

## Graphical abstract

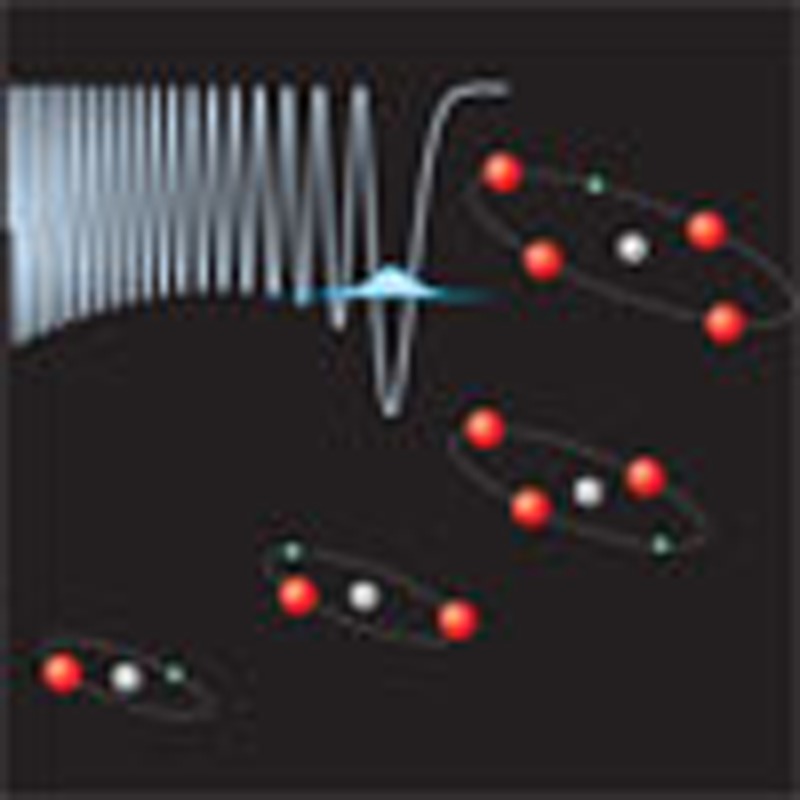

Ultralong-range Rydberg molecules, theoretically predicted in ref. 1, show very weak binding energies, similar to magneto-associated Feshbach molecules^2^,^3^, and thus require ultracold temperatures. The bond in these molecules results from the scattering of a slow Rydberg electron from a neutral atom with a negative scattering length *a* (ref. 1). The theoretical approach is based on Fermi’s original concept of the pseudopotential^4^


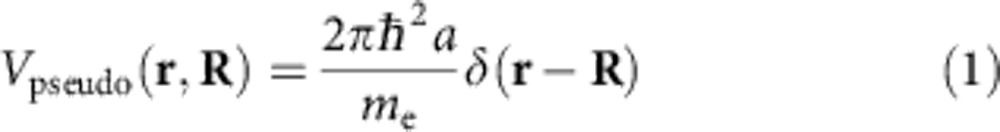


describing such a scattering event between an electron of mass *m*_e_ at position **r** and an atom at **R**. If the scattering length *a* is negative, the interaction is attractive and ground state atoms can be bound in a potential


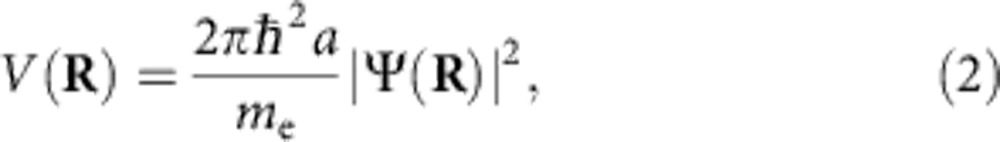


where 
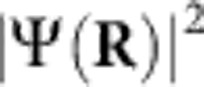
 is the local electron density. In case of ^87^Rb the theoretical value of the scattering length *a* for triplet scattering is −16.1*a*_0_ (ref. 5), where *a*_0_ is the Bohr radius. The singlet scattering length is positive and therefore does not lead to a bound state.

The vibrational ground state wavefunction belonging to the diatomic (polyatomic) molecules with the highest binding energy per atom is localized mainly at the position of the outermost lobe of the electron wavefunction, that is, near the classical turning point of the electron. Molecules containing more than one ground state atom can be described with the same formalism. Two ground state atoms inside the electron orbit essentially do not interact with each other, as the Rb atom–atom scattering length^6^ is much smaller than the mean particle distance in a dilute thermal cloud. Hence, the binding energy of an *i*-atomic molecule, *i*∈N, is (*i*–1) times larger than the binding energy of a dimer. Only when the number of ground state atoms inside the Rydberg atom becomes very large, a change in the description of the system from discrete bound states to a mean field approximation is required. In this case no individual bound states are resolved, but the Rydberg line is shifted by


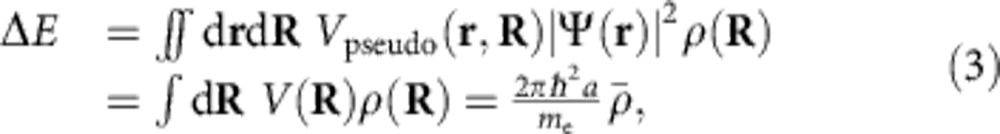


where *ρ* is the ground state atom density and 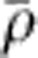 its average weighted with the probability density of the Rydberg electron. If higher order corrections to the zero-energy scattering length can be neglected, the mean shift depends only on the value of *a* and 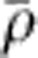.

A wide range of fascinating phenomena, starting from the coherent creation and breaking of chemical bonds^7^ to a permanent electric dipole moment in a homonuclear molecule^8^, have been already studied in the field of ultralong-range Rydberg molecules. Dimers, consisting of a single atom in the Rydberg state and one atom in the ground state, have been observed in an ultracold gas of Rb in the Rydberg *S*-state^9^, *D*-state^10^,^11^ and *P*-state^12^, and of Cs (ref. 13) in the Rydberg *S*-state. The regime, where the Rydberg electron experiences a mean shift by thousands of atoms within its orbit, has been studied at very high densities in a Bose–Einstein condensate, leading to electron–phonon coupling^14^, and in a hot vapour^15^. Here we trace the transition between the resolvable molecular lines and the mean shift regime in an ultracold cloud with a constant density by tuning only one parameter: the principal quantum number *n* of the excited Rydberg state. We probe the limit at which the binding energy per atom becomes very small, while the radius of the molecules grows beyond 10,000*a*_0_.

## Results

### Molecular spectroscopy

**Figure 1:**
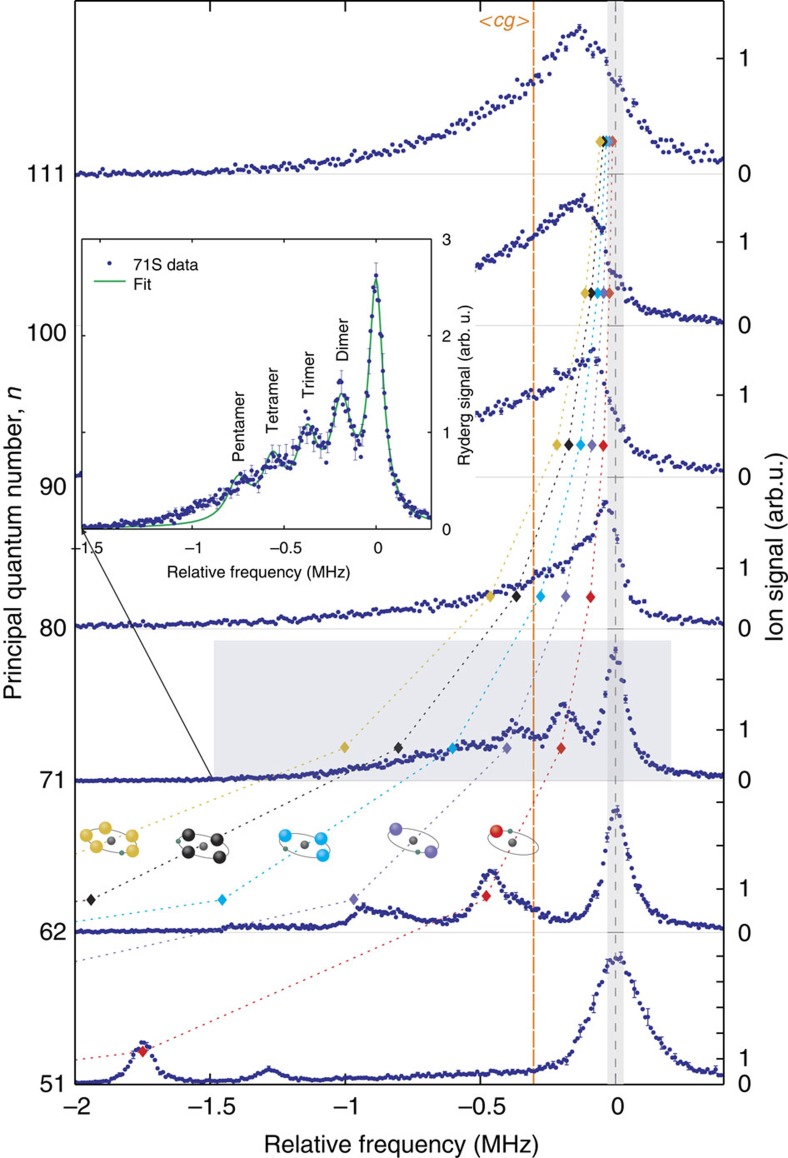
Overview of the 5*S*_1/2_ to *nS*_1/2_ excitation spectra. The dimer—polyatomic molecule transition for increasing principal quantum number is visible. The origin of the relative frequency axis corresponds to the centre of the atomic line (dashed line) for *n*≤71, where molecular lines are distinguishable. The grey shaded area between −30 and 30 kHz indicates the laser bandwidth. Spectra at *n*>71 are horizontally shifted such that their centres of gravity overlap with the mean centre of gravity 〈*cg*〉 (orange dashed line) of the first three spectra (*n*=51, 62, 71). All data were taken at similar cloud parameters, therefore the density-induced shift for all spectra is constant in the first approximation. Molecules with up to three bound ground state atoms for 62*S* and up to four for 71*S* are resolvable in the spectra. Coloured diamonds indicate the positions of the dimers (red), trimers (violet) etc. following the power law scaling of the binding energies fitted to the first three spectra. In the inset, the molecular spectrum for the *n*=71 Rydberg state is shown. A multilorentzian fit (green line), assuming a constant spacing between the molecular peaks is plotted to indicate the positions of the higher order molecular lines. The spectrum for *n*=40 is not shown, because the binding energy of the dimer is larger than the plotting range. Each spectrum is an average over 20 independent measurements with standard deviation error bars. arb. u., arbitrary unit.

**Figure 2:**
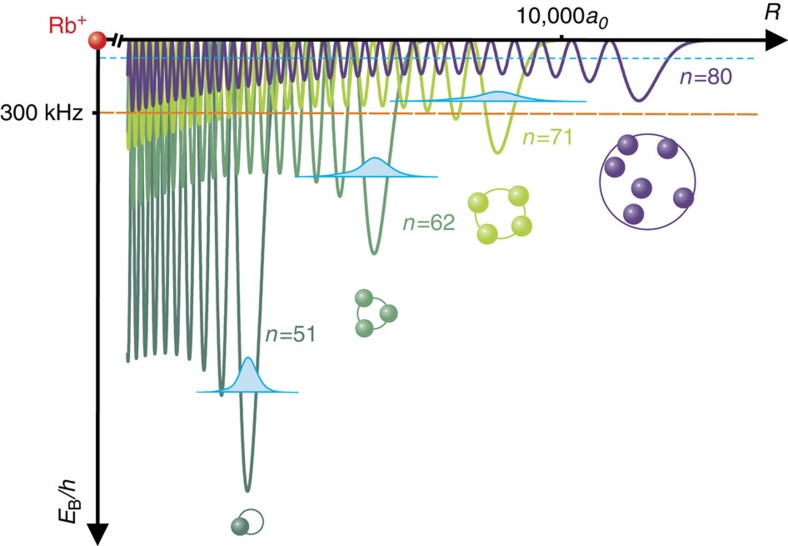
Scattering potentials for *n*=51, 62, 71 and 80 *S*-states. The potentials are calculated from equation (2). Dimers are predominantly bound in the vibrational ground state in the outermost well (probability density in light blue). At *n*=80, the mean binding energy per atom (light blue dashed line) becomes comparable to the experimental resolution and a single-molecular state localized in the outermost lobe cannot be resolved in the experiment. The spheres below illustrate the highest order polyatomic molecule observed for each principal quantum number. The orange dashed line indicates the mean shift for the experimental density. All potentials, binding energies and probability densities are plotted to scale.

In Fig. 1 excitation spectra from the 5*S*_1/2_ to the *nS*_1/2_ state, where *n* is ranging from 51 to 111, are presented. Comparison of some of the relevant molecular potentials are shown in Fig. 2. The shape of the obtained Rydberg spectra varies significantly for different *n*. For low principal quantum numbers clearly distinguishable molecular lines are present on the red side of the atomic peak, which is situated at the origin. We can identify the observed molecular lines by calculating the binding energies of dimers in the ground and the first vibrationally excited state^9^. The binding energy *E*_B_ can be directly measured as the difference between the atomic and the molecular line in the spectrum. In the spectrum of *n*=51 the peak at −1.7 MHz can be identified as a dimer, for which the ground state atom is localized in the outermost well of the molecular potential. Additionally, at −1.3 MHz an excited vibrational state^9^,^16^ is visible. For a given density, the probability to find an atom inside the electron orbit scales as 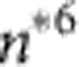 with the effective principal quantum number n*=*n*−*δ*_0_. Hence, higher order molecules are formed more likely at higher *n*. At the same time the binding energy per atom decreases and thus polyatomic molecular lines become visible in the spectra of *n*=62 and 71. At *n*=62, lines up to the tetramer together with corresponding vibrational excited states are visible. The broadening of the tetramer line may be caused by the presence of these excited states, possibly with a reduced lifetime^17^. At *n*=71 only vibrational ground states are resolved. Polyatomic molecules up to a pentamer can be identified. The size of such a molecule becomes enormous due to the Rydberg electron orbit radius reaching almost 10,000*a*_0_ (Fig. 1). Since the thermal cloud is much bigger than the Rydberg atom, the Gaussian density distribution does not play a role on a single Rydberg atom level, and the spatial arrangement of the bound ground state atoms is isotropic. For large *n* the binding energy *E*_B_ decreases until it is below the experimental resolution. This manifests in a non-resolvable shoulder and finally in an inhomogeneously broadened spectral line.

### Analysis of the spectra

The experimental binding energies can be calculated based on the molecular potential (2) and the mean shift (3). Momentum-dependent corrections to these values can be estimated using a semiclassical approximation^18^. Additionally, a *p*-wave shape resonance can cause a substantial modification of the molecular potential, leading to butterfly-type molecules^19^ and states bound by internal quantum reflection^16^. These corrections are largely negligible for high principal quantum numbers and large distances from the Rydberg core, where the relative motion of the Rydberg electron and the perturber is slow. We use corrections to the *s*-wave scattering length including terms linear in the relative momentum. As this approximation is valid for large distances from the ionic core, we restrict the analysis to the lowest bound state. The discussion of higher vibrational states can be found in ref. 16. We solve the Schrödinger equation for the ground state atom in the molecular potential using Numerov’s method to find the binding energies of the dimers. We fit the zero-energy scattering length *a* to obtain the best agreement with the measured values. In this paper we achieve the best agreement with the experimental data for −16.2*a*_0_, which is very close to the theoretically predicted value of −16.1*a*_0_ (ref. 5).

For *n*>71, where no distinct molecular lines can be identified, a mean field description is required. Furthermore, the spectral position of pure Rydberg atoms, and thus the zero position, cannot be identified directly from the signal. However it can be determined from the centre of gravity *cg* of the spectra, taken in a non-blockaded sample, which is constant for a given density. Intuitively, this result in the first approximation can be explained by the fact that while the mean potential depth 
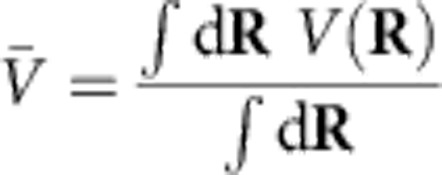
 averaged over the volume of the Rydberg atom decreases with the effective principal number as 
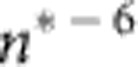
, the probability to find an atom inside the Rydberg electron orbit increases with 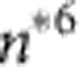. In the experiment all data were taken at a fixed density. Thus, in our analysis we overlap the centre of gravity of the spectra at *n*≥71 with the mean 〈*cg*〉=−300 kHz determined at low principal quantum numbers. Doing so we can identify the zero position in the top panels of Fig. 1. Assuming the scattering length *a* to be constant, we determine the effective density to be 3 × 10^12^ cm^−3^, which is close to the peak density obtained from a Gaussian fit to absorption images of the thermal cloud. This indicates that molecules are most likely created in regions of high density. Note that the power broadening would not effect the centre of gravity of the measured spectra. Only for *n*≤80 the highest signal originates from pure Rydberg atoms. Already for *n*=80 there is on average one ground state atom inside the electron orbit, leading to a high probability to excite dimers instead of pure Rydberg atoms. On average there are four atoms inside the 100*S* electron orbit and eight for the 111*S* state. Therefore, the atomic line is suppressed, while the molecular contribution to the overall signal increases. At the same time the molecular lines are not resolvable in the experiment any more, because of their very low binding energies. For even higher principal quantum numbers the shape of the spectrum is expected to become Gaussian, with the maximum at the position of the mean shift (〈*cg*〉 in Fig. 1) (refs 14, 20).

**Figure 3:**
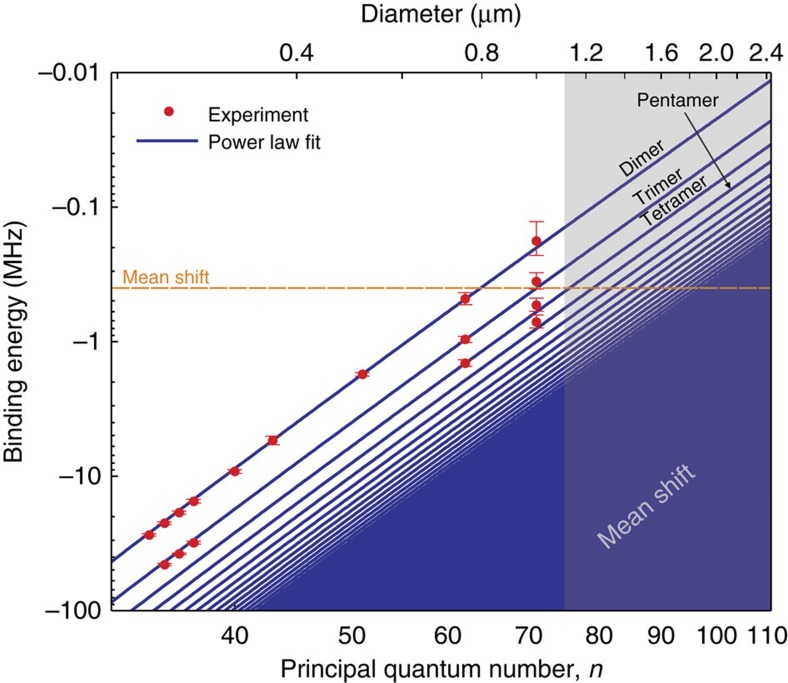
Measured binding energies versus principal quantum number and diameters of the molecules. The data for *n*≤37 are taken from ref. 9. For *n*=40, 43, 51, the frequency range chosen in the experiment was too small to photoassociate molecules with larger binding energies than a dimer. The power law 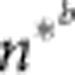 (blue lines) is fitted to the measured binding energies (red points) of the dimers and multiplied by factors *i*–1, *i*∈N, (*i*=2 for a dimer). The grey shaded area indicates the mean shift regime, which starts at around *n*=75, which can also be seen in Fig. 1. At this principal quantum number, the binding energy of the dimer becomes smaller than the experimental resolution. Calculated values of *E*_B_ are not shown in the plot, because they are hardly distinguishable from the experimental data on this scale. The orange dashed line indicates the calculated 〈*cg*〉 from the measured spectra (Fig. 1). The error bars are determined as the standard deviation of the fit.

The binding energies of all observed molecules are plotted in Fig. 3. From the extrapolated molecular binding energies the transition from the few particle description of discrete bound states to a mean field shift becomes visible. A power law fitted to the *n*≥40 data shows a scaling with the effective principal quantum number n* to the power of −6.26±0.12, close to the value of −6, expected from the size scaling argument. The deviation can be explained by the dependence of the scattering length on the relative momentum and the fact that with increasing principal quantum number the shape of the outermost well of the molecular potential changes. Taking the corresponding zero-point energy and momentum-dependent corrections to the scattering length into account, we obtain an exponent of −6.37, which is in very good agreement with our experimental data. Contributions of higher order partial waves and *p*-wave shape resonances to the molecular potential can be neglected, as the kinetic energy associated with the relative motion of two scattering partners is small in the region of interest.

## Discussion

The obtained experimental results have strong implications, for example, in the field of Rydberg quantum optics. The density-dependent dephasing resulting from the existence of many molecular lines within the Rydberg line envelope sets a fundamental limit for the number of atoms inside the blockade radius, that is, the optical thickness, for *n*>80. This fact is of importance for every experiment taken at high principal quantum numbers and high densities, in particular for quantum optics experiments in ultracold clouds^21^,^22^,^23^,^24^. In many experiments based on electromagnetically induced transparency using a Rydberg state, the figure of merit is high optical depth per Rydberg blockade volume, which is set by the width of the electromagnetically induced transparency window^25^. Assuming a typical linewidth of 1 MHz, density-induced decoherence becomes relevant at the density 10^13^ cm^−3^ (ref. 23). Therefore, this effect has to be taken into account in the experimental work and proposals, as in practice it will limit the Rydberg blockade.

Nonetheless, Rydberg molecules offer a unique tool to address few-body subsystems with control on a single-particle level by changing the detuning of the excitation laser light. The number of constituents of a molecule and the interaction strength can also be varied independently by changing the density and the principal quantum number of the excited Rydberg atom. Furthermore, the analysis of the relative strength of the molecular lines opens up the possibility to measure correlations in a bosonic gas. In high-density gases and for low principal quantum numbers, where the size of the Rydberg atom is comparable to the de Broglie wavelength, extracting the *g*^(2)^ correlation function^26^ of thermal and Bose-condensed gases is feasible. In addition to previous measurements^27^,^28^ also higher order correlation functions can be studied using polyatomic molecules.

## Methods

### Experiment

We perform the experiment in a magnetically trapped ultracold cloud of ^87^Rb atoms in the 5*S*_1/2_, *F*=2, *m*_F_=2 ground state with typical temperatures of 2 μK and densities on the order of 10^12^ cm^−3^. Detailed information about the setup can be found in ref. 29. After the preparation of the ultracold cloud, we excite the atoms in a two-photon process via the 6*P*_3/2_ state to the *nS*_1/2_ Rydberg state (quantum defect *δ*_0_=3.1311807(8) (ref. 30)). The detuning from the intermediate state in the two-photon transition is 80 MHz. The light driving the lower transition is sent to the experiment in 50 μs pulses with a repetition rate of 167 Hz. During the sequence the light driving the upper transition is on constantly. Both laser beams have beam waists of 0.5 mm. After the excitation we field-ionize the Rydberg atoms and collect the ions on a microchannel plate detector. In a single atomic cloud we perform typically 400 cycles of Rydberg excitation and detection while scanning the frequency of the infrared laser light. We choose long excitation pulses of 50 μs to not be limited by Fourier broadening. Taking further into account the Doppler broadening this results in an experimental resolution with full-width at half-maximum value of 100 kHz. To obtain the best visibility while changing the principal quantum number of the excited Rydberg state, we adjust the power of the laser driving the lower transition to account for power broadening, therefore we maintain a two-photon Rabi frequency Ω below 600 Hz. Only the spectrum of *n*=51 was taken with higher laser power (Ω≈750 Hz) and thus in this case the atomic line is slightly broadened. With these Rabi frequencies the calculated blockade radii for the measured states are ranging from 7 μm (51*S*) to 28 μm (111*S*); for comparison the 3*σ* radius of the thermal cloud is around 0.4 mm. All the measurements were taken in the dilute regime for Rydberg atoms.

## Author contributions

The experiment was conceived by A.G., A.T.K., R.L., S.H. and T.P., and carried out by A.G. and A.T.K.; data analysis was accomplished by A.G. and A.T.K.; numerical calculation was performed by A.G. and J.B.B., and A.G. wrote the manuscript with contributions from all authors.

## Additional information

**How to cite this article:** Gaj, A. *et al.* From molecular spectra to a density shift in dense Rydberg gases. *Nat. Commun.* 5:4546 doi: 10.1038/ncomms5546 (2014).
